# Desmoglein 2 is a substrate of kallikrein 7 in pancreatic cancer

**DOI:** 10.1186/1471-2407-8-373

**Published:** 2008-12-17

**Authors:** Vishnu C Ramani, Leah Hennings, Randy S Haun

**Affiliations:** 1 Department of Pathology, Winthrop P. Rockefeller Cancer Institute, University of Arkansas for Medical Sciences, Little Rock, Arkansas, USA

## Abstract

**Background:**

In a previous report we have demonstrated that the chymotryptic-like serine protease kallikrein 7 (*KLK7*/hK7) is overexpressed in pancreatic cancer. In normal skin, hK7 is thought to participate in skin desquamation by contributing in the degradation of desmosomal components, such as desmogleins. Thus, the ability of hK7 to degrade desmogleins was assessed and the effect of hK7 expression on desmoglein 2 was examined in cultured pancreatic cancer cells.

**Methods:**

The expression of Dsg1, Dsg2, and Dsg3 in pancreatic tissues was examined by immunohistochemistry and their expression in two pancreatic cancer cell lines, BxPC-3 and Panc-1, was determined by western blot analysis. The ability of hK7 to degrade Dsg1 and Dsg2 was investigated using *in vitro *degradation assays. BxPC-3 cells stably transfected to overexpress hK7 were used to examine the effect of hK7 on cell-surface resident Dsg2.

**Results:**

The levels of immunoreactive Dsg1 and Dsg2 were reduced in pancreatic adenocarcinomas compared with both normal pancreatic and chronic pancreatitis tissues. Among the desmosomal proteins examined, Dsg2 exhibited robust expression on the surface of BxPC-3 cells. When hK7 was overexpressed in this cell line, there was a significant increase in the amount of soluble Dsg2 released into the culture medium compared with vector-transfected control cells.

**Conclusion:**

A reduction in the amount of the cell adhesion components Dsg1 and Dsg2 in pancreatic tumors suggests that loss of these desmosomal proteins may play a role in pancreatic cancer invasion. Using *in vitro *degradation assays, both Dsg1 and Dsg2 could be readily proteolyzed by hK7, which is overexpressed in pancreatic adenocarcinomas. The enforced expression of hK7 in BxPC-3 cells that express significant amounts of Dsg2 resulted in a marked increase in the shedding of soluble Dsg2, which is consistent with the notion that aberrant expression of hK7 in pancreatic tumors may result in diminished cell-cell adhesion and facilitate tumor cell invasion.

## Background

Pancreatic cancer is one of the deadliest of all human cancers, resulting in more than 30,000 deaths per year in the United States alone, and continues to be a major health problem in terms of detection as well as treatment. Pancreatic cancer is highly invasive and is characterized by early metastasis. Tumor invasion and metastasis is a multi-step process involving several key cellular events [1]. Among the many events leading to tumor dissemination and metastasis, loss of intracellular adhesion is one of the earliest events [2].

Among the classes of adhesion molecules, desmosomes have been widely recognized and studied for their various roles in cell adhesion, tissue morphogenesis, and cell signaling [3]. Desmosomes, apart from being adhesive intracellular junctions, also act as a membrane anchor for intermediate filaments [4]. The core of the desmosomal adhesive complex primarily consists of desmogleins (Dsg) and desmocollins (Dsc), glycoproteins belonging to the cadherin superfamily of proteins. At least four different isoforms of desmogleins (Dsg1–4) and three different isoforms of desmocollins (Dsc1–3) have been reported thus far. As seen with many other important adhesion molecules, alterations in the expression of various members of desmosomal family of proteins have been observed in different types of cancer [5]. There is, however, a lack of complete understanding regarding the expression of desmosomal proteins in many different types of cancer and the mechanism by which cancer cells may regulate and overcome the adhesion mediated by desmosomal proteins.

One of the most well characterized mechanisms by which tumor cells can overcome adhesion mediated by intercellular adhesive molecules is by up-regulating the expression of various families of proteases that are capable of proteolyzing one or more of these cellular adhesions [6-9]. Among the various families of proteases, the kallikreins are known to play an important role in many different disease states, including cancer [10-12]. In a previous study, we have reported that kallikrein 7 (*KLK7*/hK7) is overexpressed in pancreatic adenocarcinomas and enhances pancreatic cancer cell invasion by shedding E-cadherin [13]. Human kallikrein 7 (hK7), originally named stratum corneum chymotryptic enzyme, was initially characterized from extracts of human skin and shown to play an important role in normal skin desquamation by degrading desmogleins and corneodesmosomes [14,15] along with other kallikreins [16]. However, the effects of hK7 expression on desmosomal proteins in any type of cancer, including pancreatic cancer where overexpression of *KLK7*/hK7 has been clearly established, have not been studied. Herein, we show for the first time that the overall expression levels of desmogleins 1 and 2 are lower in human pancreatic adenocarcinomas compared to chronic pancreatitis and non-malignant pancreatic tissues and that both of these desmosomal proteins are substrates for hK7. Additionally, expression of *KLK7 *in the human pancreatic adenocarcinoma cell line BxPC-3 significantly increased the amount of soluble desmoglein 2 shed from the cell surface, which correlates with the *in vitro *degradation data. These results extend the potential roles for the aberrant expression of hK7 observed in pancreatic cancer and points toward a critical role for this protease in aiding cancer invasion via its action on important cellular adhesive molecules like desmogleins.

## Methods

### Immunohistochemistry

For each antigen examined, formalin-fixed, paraffin-embedded tissue blocks from six non-malignant pancreas, six chronic pancreatitis, and six pancreatic adenocarcinoma tissues were prepared for immunohistochemical analysis. Representative hematoxylin and eosin-stained sections from each tissue were evaluated by microscopic analysis. Sections (4 μm) were deparaffinized and rehydrated in xylene followed by graded ethanol. Antigen retrieval was performed in a 95°C water bath using 10 mM citrate, pH 6.0, for 30 minutes. Endogenous peroxidase activity was quenched by hydrogen peroxide treatment followed by serum-free protein block (DakoCytomation, Carpinteria, CA). Sections were incubated with desmoglein 1, 2, or 3 antibodies (R&D Systems, Minneapolis, MN), diluted 1:75 in antibody diluent (DakoCytomation), overnight at 4°C. Immunoreactive staining was detected using a DAKO LSAB+ peroxidase system followed by hematoxylin counterstain. Using direct ELISA and western blot analyses to examine the specificity of the desmoglein antibodies, the supplier reports that the Dsg1 antibody shows approximately 5% cross-reactivity with rhDesmoglein-2 and the Dsg2 antibody shows less than 1% cross-reactivity with rhDesmoglein-1. The acquisition and use of archived, paraffin-embedded human tissue samples in this study were reviewed and approved by the UAMS Human Research Advisory Committee.

### Quantitation of immunohistochemical staining

To numerically analyze the immunohistochemical staining, virtual slides were created from the stained samples after scanning each specimen using an Aperio ScanScope scanning system (Aperio Technologies, Vista, CA). The ScanScope generated true color digital images of each stained sample, which were viewed using Aperio Imagescope v.6.25 software. The algorithm for determining the intensity of membrane-specific staining provided by the manufacturer was optimized and used to calculate the staining intensity and percent target labeled for each sample by digitally analyzing the color intensity. A color markup image for each slide was obtained based on membrane staining intensity. The output was viewed as determinations of staining intensity ranging from 0+ to 3+ to correlate with conventional manual scoring methods and the percentage of cells stained for either Dsg1 or Dsg2 was averaged for non-malignant, pancreatitis, pancreatic cancer samples and represented as a stacked column graph.

### *In vitro *degradation assays

Recombinant, pro-hK7 (100 μg/mL) (R&D Systems) was proteolytically activated using 10 μg/ml thermolysin (R&D Systems) and its proteolytic activity was verified using a fluorogenic substrate, ES002 (R&D Systems), according to manufacturer's instructions. Thermolysin-activate hK7 (25 ng) was then added to 100 ng of either recombinant Dsg1 or Dsg2 (R&D Systems). The mixtures were split into two fractions and thermolysin activity was inhibited in both the fractions with 0.4 mM (final concentration) phosphoramidon (Sigma-Aldrich, St. Louis, MO). As a control, chymostatin was added (0.28 mM final concentration) to one fraction to inhibit hK7 activity. All the samples were incubated at 37°C and at 30 minute intervals an aliquot was removed from each mixture, added to SDS-PAGE sample buffer, heated at 95°C for 5 minutes, and stored at 4°C for further analysis. At the end of the incubation period, all the samples were separated using 4–12% gradient Bis-Tris NuPAGE gels (Invitrogen, Carlsbad, CA) and transferred to PVDF membranes. The membranes were blocked with a 5% non-fat milk solution in Tris-buffered saline, pH 7.4, containing 0.1% Tween-20 (TTBS) then incubated overnight at 4°C with either Dsg1 or Dsg2 antibody (R&D Systems) diluted 1:1250 with TTBS. After washing with TTBS, the blots were incubated for 1 hour at room temperature with HRP-conjugated polyclonal anti-goat immunoglobulins (DakoCytomation) diluted 1:2000 in TTBS. The blots were washed with TTBS and visualized by chemiluminescence using ECL plus reagent (GE Healthcare, Piscataway, NJ) and a ChemiDoc XRS image documentation system and Quantity One analysis software (Bio-Rad, Hercules, CA).

### Immunoblotting for Dsg1 and Dsg2 in pancreatic cancer cell lines

Human pancreatic adenocarcinoma cell lines Panc-1 and BxPC-3 (American Type Culture Collection, Manassas, VA) were grown in 10-cm plates at 37°C in a 5% CO_2 _incubator in Dulbecco's modified Eagle's medium (DMEM) (Invitrogen) containing 10% fetal bovine serum (Atlanta Biologicals, Norcross, GA). Confluent monolayers were harvested by scraping, washed twice with phosphate-buffered saline (PBS), and lysed with RIPA buffer (1 mM EDTA, 1% NP-40, 0.5% deoxycholate, 0.1% SDS in PBS) containing Complete protease inhibitor cocktail (Roche, Indianapolis, IN). The cell lysates were sonicated on ice and centrifuged at 13,000 rpm to remove any cell debris and the protein concentration of each lysate was determined using a bicinchoninic acid assay (BCA) (Sigma-Aldrich). Two samples of each lysate (50 μg of total protein) were resolved by SDS-PAGE and transferred to PVDF membrane. The membrane was cut and probed for Dsg1 or Dsg2 (upper portion) and GAPDH (lower portion) by western blot.

### Immunocytochemistry

Panc-1 and BxPC-3 cells (3 × 10^4^) were seeded in Lab-Tek II four-chamber glass slides (Nalge Nunc, Naperville, IL). Confluent monolayers were washed twice with PBS then fixed with 4% paraformaldehyde in PBS at room temperature for 20 minutes. The fixed cells were washed with PBS, permeabilized with -20°C chilled methanol, washed with PBS, and incubated with serum-free protein block (DAKO). The cells were then incubated overnight at 4°C with a polyclonal Dsg2 antibody (diluted 1:75), washed with PBS containing 0.1% Triton X-100 (TPBS), and incubated with a fluorescein isothiocyanate (FITC)-conjugated rabbit anti-goat antibody (DakoCytomation) for 2 hours at room temperature. Cells were washed with TPBS, mounted with Vectashield mounting medium (Vector Laboratories, Burlingame, CA), and visualized using an Olympus BX41 fluorescent microscope (Olympus USA, Melville, NY). Images were captured with a SPOT-RT digital camera and software (Diagnostic Instruments, Sterling Heights, MI).

### Detection of soluble desmoglein 2

One million vector-transfected or *KLK7*-expressing BxPC-3 cells [17] were seeded in 10-cm dishes. Growth medium from confluent monolayers was removed, cells were washed twice with serum-free medium (SFM), and then incubated in SFM containing 1.5 μg/ml puromycin for 24 hours. The conditioned medium was removed and centrifuged at 1500 rpm for 5 minutes at 4°C to remove detached cells and debris and then concentrated using Amicon Ultra-4 centrifugal filter units, 10-kDa nominal molecular weight limit (Millipore, Billerica, MA), according to manufacturer's instructions. The cell monolayers were harvested by trypsinization and single-cell suspensions were prepared and enumerated using a Z1 Coulter counter (Beckman Coulter, Fullerton, CA). The total number of cells per volume of final concentrate for each sample was calculated and the volume of concentrate corresponding to 10^6 ^cells for each sample was mixed with SDS sample buffer and heated at 95°C for 5 minutes. The samples were then separated using a NuPage 4–12% gradient polyacrylamide gel (Invitrogen) and transferred to a PVDF membrane. The immunoblot was blocked with a 5% non-fat milk solution in TTBS then incubated overnight with anti-Dsg2 polyclonal antibody (diluted 1:1250) (R&D Systems). After washing with TTBS, the blot was incubated with horseradish peroxidase-(HRP) conjugated anti-goat immunoglobulins (diluted 1:2000) (DakoCytomation) and soluble Dsg2 (sDsg2) was visualized by chemiluminescence using ECL plus reagent and a ChemiDoc XRS image documentation system. The amount of sDsg2 released into the conditioned medium of each sample was quantified using Quantity One image analysis software. The mean intensity of three independent experiments was determined and analyzed for statistical significance using an unpaired, 2-tailed *t*-test (GraphPad Software, San Diego, CA).

## Results

### Intensity of membrane staining for desmosomal proteins Dsg1 and Dsg2 is lower in human pancreatic cancer compared to normal pancreas and chronic pancreatitis

**Figure 1:**
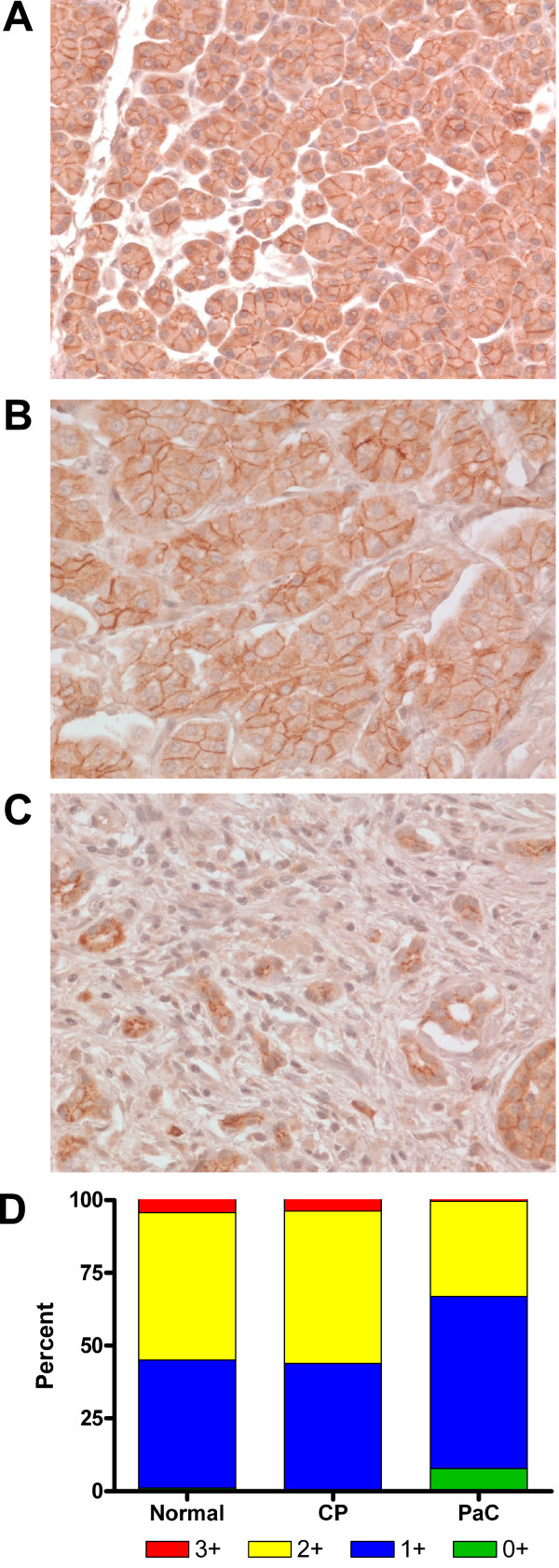
**Desmoglein 1 is present in lower levels in pancreatic cancer compared to normal and chronic pancreatitis tissue**. Representative pancreatic tissue sections stained for Dsg1 showed high membrane staining in (A) normal pancreas, (B) chronic pancreatitis (CP), and (C) weaker staining in pancreatic adenocarcinoma (PaC) samples. Original magnification x400. (D) Staining intensity of the cell membranes was quantitated (see Methods), categorized into intensity ranges from 0+ to 3+, and the average percentage of cells in each group was determined and represented in a stacked graph for each tissue type (n = 6).

**Figure 2:**
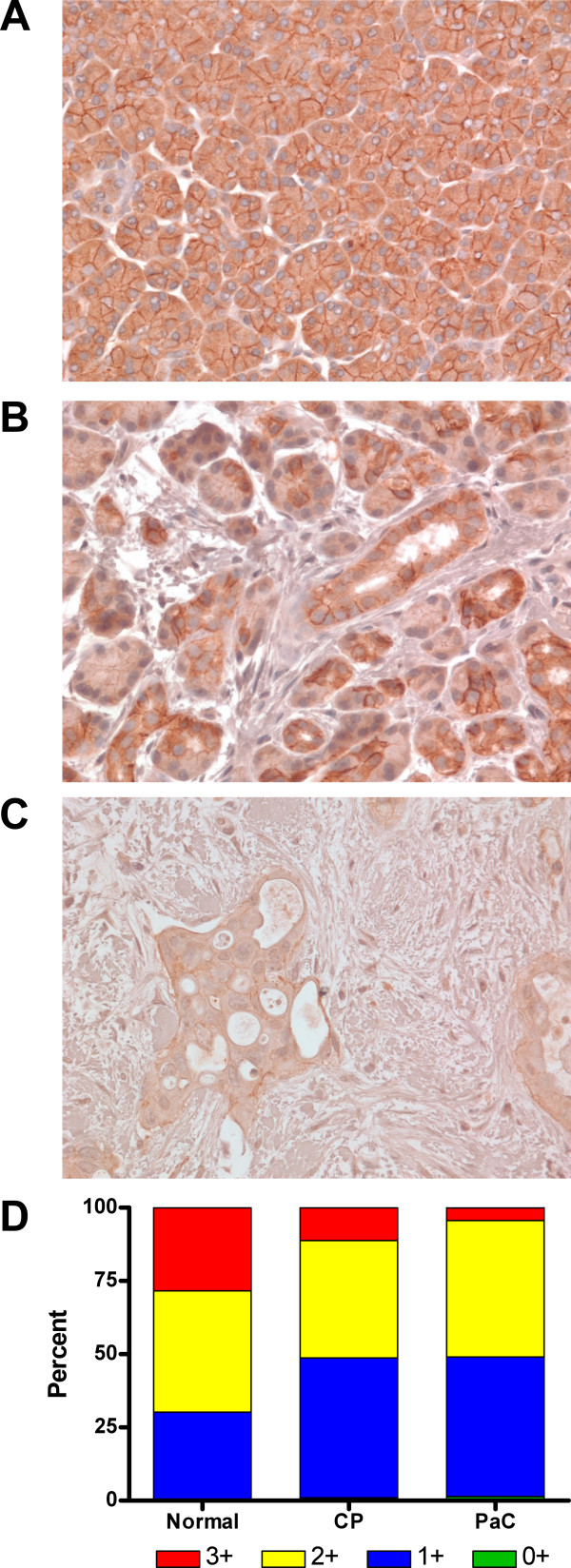
**Desmoglein 2 staining is lower in pancreatic cancer cell membranes compared to normal and chronic pancreatitis tissue**. Representative pancreatic tissue sections stained for Dsg2 showed high levels of membrane staining in (A) normal pancreas and (B) chronic pancreatitis (CP) samples. Membrane staining was much weaker in (C) pancreatic adenocarcinoma (PaC) samples. Original magnification x400. (D) Staining intensity of the cell membranes was quantitated (see Methods), categorized into intensity ranges from 0+ to 3+, and the average percentage of cells in each group was determined and represented in a stacked graph for each tissue type (n = 6).

Six independent sections of normal human pancreas, chronic pancreatitis, or pancreatic adenocarcinoma tissue were stained for desmogleins 1, 2, and 3. Desmogleins 1 and 2 were clearly present in all of the normal pancreatic tissue samples examined and showed intense staining at the cell-cell borders (Fig. 1A and 2A, respectively). In contrast, Dsg3 was not detected in any of the pancreatic tissues (data not shown). The chronic pancreatitis samples showed staining intensity and distribution similar to the normal pancreatic samples for Dsg1 (Fig. 1B) and Dsg2 (Fig. 2B). All the pancreatic cancer samples displayed an intense desmoplastic response, the synthesis and deposition of collagenous material by stromal myofibroblasts surrounding the adenocarcinoma, and the membrane staining for both Dsg1 (Fig. 1C) and Dsg2 (Fig. 2C) was distinctly weaker in most of the cancer samples analyzed. The correspondence between increased hK7 expression in pancreatic tumors and loss of Dsg2 is highlighted in the immunohistochemistry studies we have performed. For example, the tumor section stained for Dsg2 (Figure 2C, this study), showing a loss of Dsg2 immunoreactivity, is from the same tissue used to demonstrate increased hK7 expression in pancreatic tumors (Figure 2A, ref. [13]).

To further quantify the intensity of membrane staining for Dsg1 and Dsg2 in the different tissue samples, the stained slides were scanned with an Aperio ScanScope and areas of cellularity in the digital images were selected by a pathologist (L.H.) and processed to calculate the staining intensity and percent label for each sample. The average percentage of cells stained for Dsg1 within each intensity category (e.g., 1+, 2+) was nearly equal between the normal pancreatic tissues and chronic pancreatitis samples (Fig. 1D). The pancreatic cancer samples, however, showed an almost complete loss of cells displaying intense (3+) Dsg1 staining and a marked decrease in cells with 2+ intensity with a concomitant increase in cells with weak (1+) or absent (0+) Dsg1 staining.

An examination of Dsg2 immunoreactivity revealed that the most intense staining (3+) was observed in the normal pancreatic tissues (28.4%), although the chronic pancreatitis samples (11.2%) still showed a higher average percentage of cells with 3+ intensity compared to the pancreatic adenocarcinoma samples (4.3%) (Fig. 2D). The average number of cells with 2+ intensity of staining was nearly equal in the normal and chronic pancreatitis samples and somewhat higher in pancreatic cancer samples, while the number of cells with (1+) intensity was higher in chronic pancreatitis (47.8%) and pancreatic cancer (47.7%) samples compared with the normal pancreatic tissues (30%). Although the percentage of cells lacking demonstrable Dsg2 staining (0+) increased with the severity of disease (i.e., pancreatic cancer > chronic pancreatitis > normal pancreas), the numbers were modest in all three tissue types. (Fig. 2D). Thus, overall the average intensity of membranous desmoglein staining was decreased in adenocarcinomas compared to normal pancreas and pancreatitis samples.

### hK7 cleaves both recombinant Dsg1 and Dsg2 *in vitro*

**Figure 3:**
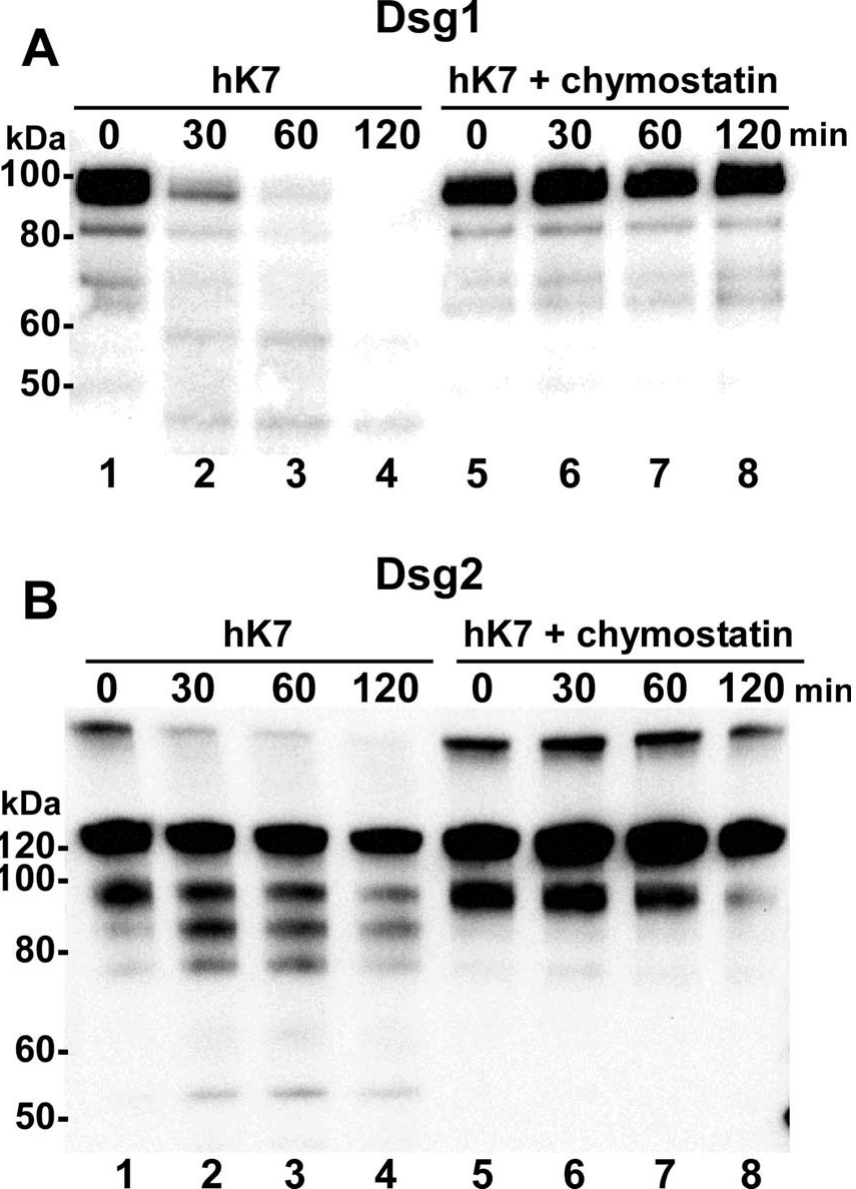
**Desmoglein 1 and 2 are substrates for hK7 *in vitro***. Thermolysin-activated hK7 was incubated with recombinant Dsg1 (A) or Dsg2 (B) for the indicated times in the absence (lanes 1–4) or presence (lanes 5–8) of the hK7 inhibitor chymostatin. Reaction products were separated by SDS-PAGE and visualized by western blot using either anti-Dsg1 or anti-Dsg2 antibodies. Sizes of protein markers are indicated on the left.

To determine whether hK7 could participate in decreasing the levels of Dsg1 and Dsg2 through proteolytic cleavage, *in vitro *degradation assays were performed using recombinant hK7 and desmosomal proteins. Upon incubation with thermolysin-activated hK7, recombinant Dsg1 was rapidly degraded with little detectable protein remaining after 60 minutes (Fig. 3A, left). Recombinant Dsg2 was also cleaved by hK7 in a time-dependent manner, however, in contrast to the complete proteolysis of Dsg1, the cleavage of Dsg2 resulted in distinct proteolytic fragments (Fig. 3B, left). All of the degradation assays were performed in the presence of phosphoramidon, which inhibits the thermolysin used to activate hK7. To verify that the degradation of the desmogleins was due to hK7 activity and not due to any residual activity of thermolysin, a parallel set of assays was performed that included chymostatin, an oligopeptide that inhibits serine proteases with chymotrypsin-like substrate specificity (Figs. 3A and 3B, right). Proteolysis of both the desmosomal proteins by hK7 was effectively inhibited in the presence of chymostatin.

### Levels of desmogleins in human pancreatic cancer cells

**Figure 4:**
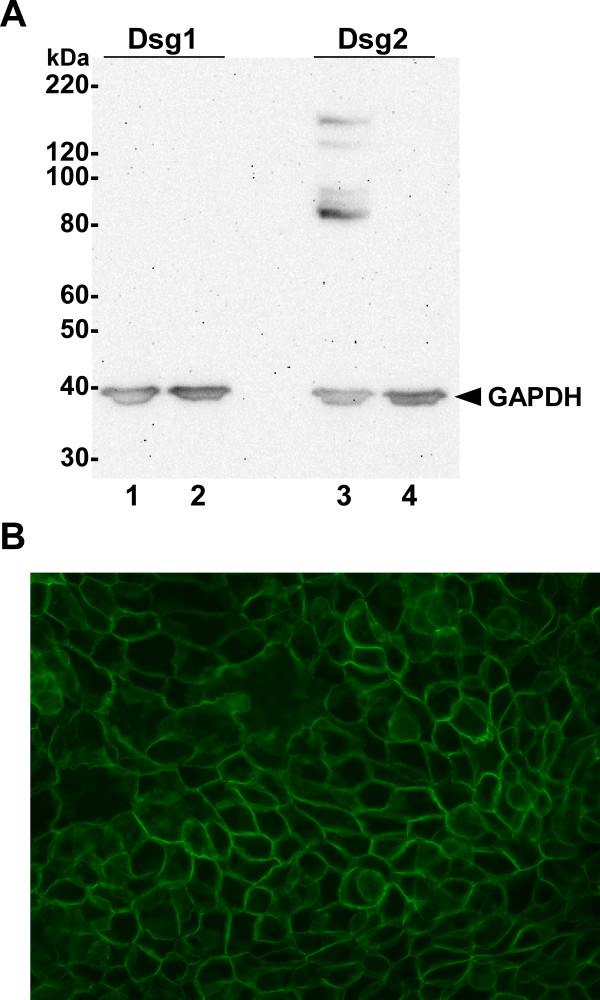
**Expression of desmogleins in human pancreatic adenocarcinoma cell lines**. (A) Equal amounts of total protein from whole cell lysates of human pancreatic cancer cell lines BxPC-3 (lanes 1 and 3) and Panc-1 (lanes 2 and 4) were separated by SDS-PAGE and visualized by western blot using anti-Dsg1 and anti-Dsg2 antibodies, as indicated. GAPDH levels were monitored as a loading control (*arrowhead*). Sizes of protein markers are indicated on the left. Appreciable levels of Dsg2 were detected in BxPC-3 cells, but not in Panc-1 cells. Dsg1 was not detected in either of the cell lines. (B) Immunocytochemistry of Dsg2 in BxPC-3 cells using a FITC-conjugated secondary antibody showed specific cell membrane localization. Original magnification x400.

To identify pancreatic cancer cell lines that could be used to examine hK7-dependent cleavage of desmosomal proteins in a cell-based system, cell lysates were prepared from Panc-1 and BxPC-3 cell lines and assessed for the levels of Dsg1 and Dsg2 by western blot analysis. Dsg1 was not detected in either of the cell lines (Fig. 4A, lanes 1 and 2), whereas Dsg2 was readily detected in BxPC-3 but not Panc-1 cell lysates (Fig. 4A, lanes 3 and 4, respectively). Due to the significant expression of Dsg2 in BxPC-3 cell lysates, immunocytochemical staining was performed on intact cells to determine its cellular localization and distribution. The staining revealed a robust cell-cell border distribution similar to the pattern seen in epithelial tissues (Fig. 4B).

### Effect of hK7 expression on Dsg2 in BxPC-3 pancreatic cancer cells

**Figure 5:**
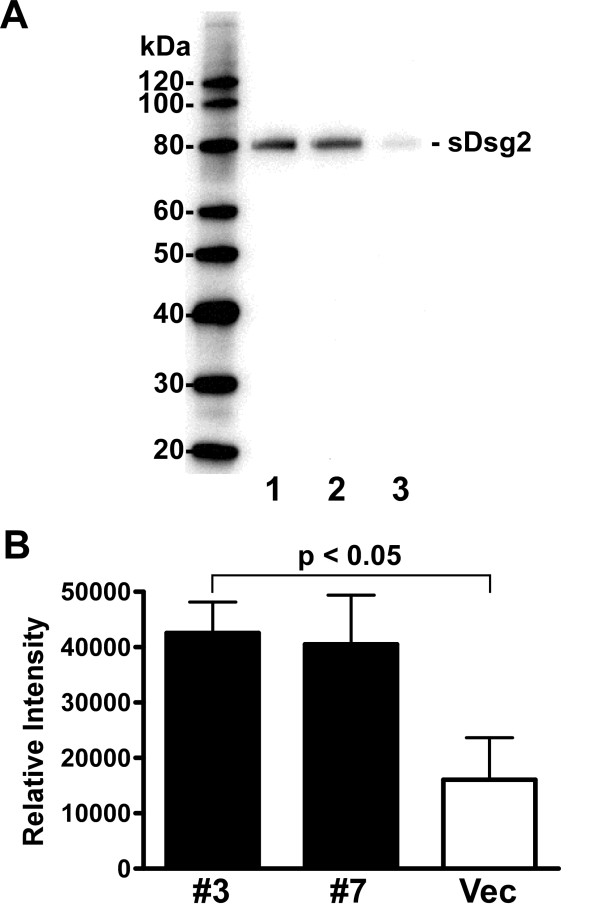
**Levels of soluble Dsg2 in conditioned medium of KLK7-expressing BxPC-3 cells are higher than in vector-transfected control cells**. (A) Levels of soluble Dsg2 (sDsg2) in concentrated conditioned medium from hK7-expressing clones BxPC-3/hK7 clone#3 (lane 1) and clone#7 (lane 2) and vector-transfected clone BxPC-3/Vec (lane 3) were determined by western blot. Sizes of protein markers are indicated on the left. (B) The relative amount of the 80-kDa sDsg2 immunoreactive product was determined for KLK7-transfected (*solid bars*) and vector-transfected (*open bar*) BxPC-3 cells by densitometric analysis using Quantity One software and the average relative intensity is depicted. Vertical bars represent SEM (n = 3).

Since BxPC-3 cells exhibit significant amounts of Dsg2, but do not express hK7 (data not shown), these cells were utilized to investigate the effects of enforced hK7 expression on Dsg2 in a cell-based system. BxPC-3 cells were transfected with a *KLK7*-expression construct or empty vector and stable transfectants were generated [17]. To obviate clonal effects, two independent *KLK7*-expressing clones, BxPC-3/hK7 clone#3 and BxPC-3/hK7 clone#7, were analyzed and compared to a vector-transfected control clone, BxPC-3/Vec. To examine the cleavage of Dsg2 from the cell surface in the presence of hK7, concentrated conditioned medium from hK7-expressing and vector-transfected control cells was prepared and the level of soluble Dsg2 was determined by western blot. Both of the hK7-expressing clones examined displayed higher levels of an 80-kDa soluble Dsg2 product compared to the vector-transfected clone (Fig. 5); thus supporting the notion that aberrant expression of hK7 may lead to the cleavage of Dsg2 from the cell surface.

## Discussion

Cell-cell adhesion and cell-extracellular matrix adhesion play a critical role as limiting factors during the early steps of invasion [1,18,19]. Various classes of cell adhesion molecules, including desmosomes, are affected in different types of cancer and tumor cells can regulate the expression and function of these adhesive molecules via various mechanisms [20,21]. Components that comprise these intercellular junctions play a critical role in determining the function and strength of these adhesive complexes [22]. Desmogleins and desmocollins, along with plakoglobin, desmoplakin, and plakophilin, represent the essential and minimum number of components that can form an intact functional desmosome [23,24]. Desmogleins, which form an integral part of the desmosomal adhesive core, are affected in many types of cancer. Various desmoglein proteins have been shown to be downregulated in urothelial carcinoma [25], oral squamous cell carcinoma [26,27], esophageal squamous cell carcinoma [28], and squamous intraepithelial lesions of the cervix [29]. Desmosomal constituents, like Dsg2, have been demonstrated to be expressed abnormally in gastric cancer [30] and its decreased expression has been associated with poorer prognosis for diffuse-type gastric cancer [31]. However, a clear understanding of the expression levels of desmogleins and the possible mechanisms leading to their decreased expression in other types of cancer, like pancreatic cancer, is still lacking.

Kallikrein 7, a chymotryptic-like serine protease that is prevalent in the stratum corneum, has been shown to directly cleave corneodesmosomes and desmosomes *in vitro *[32]; thus implicating its participation in desquamation in normal human skin [33]. Studies have also shown that hK7 is overexpressed in squamous cervical cancerous cells [34], ovarian tumor cells [35,36], cervical adenocarcinomas [37], and in lung cancer [38]. Moreover, recently we have further demonstrated that *KLK7*/hK7 is overexpressed in pancreatic adenocarcinomas [13]. However, the ability of hK7 expressed in any of these cancers to act upon desmosomal proteins, like desmogleins, which are substrates for hK7 in normal physiology, has never been explored.

Based on the aberrant expression of hK7 in pancreatic cancer, in this study we sought to determine whether the desmogleins may be substrates of hK7 in pancreatic cancer. The expression and distribution of individual desmosomal cadherins is tissue specific [39]. For example, Dsg2 is ubiquitously expressed in all tissues containing desmosomes [40], whereas Dsg1 and Dsg3 are mainly restricted to stratified squamous epithelia [41]. In the epidermis, Dsg1 is expressed throughout the upper differentiating cell layers, while Dsg3 is restricted to basal keratinocytes [41]. Thus, as a positive control we stained sections of normal human skin with the Dsg1 antibody used to detect its expression in pancreatic tissues (Additional file 1). Consistent with previous findings [42], Dsg1 was detected throughout the epidermis from superficial to basal layers. In recent studies in mice, the presence of *DSG1 *and *DSG3 *mRNAs have been reported in many epithelial organs [41]. As a first step, we surveyed human pancreatic tissue samples representing non-malignant, chronic pancreatitis, and pancreatic cancer specimens for the expression of desmoglein proteins by immunohistochemistry. Upon analysis, both desmogleins 1 and 2 showed intense membrane staining in normal pancreas and chronic pancreatitis samples. The intensity of staining for both these desmosomal proteins, however, was qualitatively much weaker in pancreatic cancer tissues. To quantitate the intensity of the membrane staining in a more objective manner, the stained sections were scanned and the digital images processed to produce a numerical representation of the staining intensity and stratification into groups typically reported in a manual analysis by a pathologist (e.g., 3+ staining). This analysis revealed that the average percentage of cells within each intensity group was similar for Dsg1 between the non-malignant and chronic pancreatitis samples, but that the percentage of more intensely stained cells for Dsg1 decreased markedly in the pancreatic cancer samples. In contrast, with Dsg2 the average number of cells with intense staining (3+) was markedly higher in non-malignant pancreas samples compared to either of the other two diseased conditions. With both Dsg1 and Dsg2, there was a pronounced shift toward the lower intensity categories in the pancreatic cancer samples. Thus, there appeared to be a clear inverse correlation between the expression of hK7 and the membrane intensity of desmosomal proteins in pancreatic samples from diseased and non-diseased conditions. No appreciable Dsg3 immunoreactivity could be detected in any of the pancreatic tissue samples. Since the ability of the antibodies used to detect Dsg3 by immunohistochemistry was verified using sections of normal human skin as a positive control (data not shown), these findings indicate that Dsg3 is not expressed at detectable levels in human pancreas.

To provide more direct evidence for a relationship between hK7 expression and the decreased intensity of desmogleins observed by immunohistochemistry, *in vitro *substrate degradation assays were performed. In support of an association between hK7 and the desmogleins, hK7 was able to proteolyze both Dsg1 and Dsg2. Proteolysis of Dsg1 resulted in a rapid degradation of recombinant Dsg1 into fragments undetectable by western blot. Interestingly, incubation of Dsg2 with hK7 resulted in cleavage of Dsg2 into distinct fragments rather than indiscriminant degradation. As further evidence of the specificity of hK7, we have tested other potential substrates under similar conditions. For example, when laminin was incubated with activated hK7, no significant cleavage was observed even after 24 hours (Additional file 2); thus, not all proteins are subject to hK7-directed proteolysis.

To identify a pancreatic cancer cell-based model to further examine the proteolysis of desmogleins by hK7, cell lysates from two pancreatic cancer cell lines, BxPC-3 and Panc-1, were screened for Dsg1 and Dsg2 by western blot analysis. This revealed that only Dsg2 was expressed in appreciable levels in BxPC-3 cells. Since BxPC-3 cells do not express hK7, we used these cells to mimic the aberrant expression of *KLK7 *observed in pancreatic tumors by expressing high levels of hK7 through stable transfection. In this manner, the effects on Dsg2 in cells with and without *KLK7 *expression could be compared, which could yield insights into changes in desmosomal proteins upon hK7 expression. In BxPC-3 cells expressing hK7, a marked increase in the shedding of an 80-kDa sDsg2 fragment was observed compared to a vector-transfected control. These findings suggest that decreased levels of desmosomal proteins seen in pancreatic cancer may result from the action of hK7 overexpressed in these tumors.

A comparison of *KLK7*-transfected and vector-transfected pancreatic cancer cells reveals that hK7 expression alters cell morphology (Additional file 3), with cells expressing hK7 exhibiting a more flattened, less refractive appearance and reduced cell-cell contacts. Using *in vitro *assays, we have demonstrated that hK7 can cleave both E-cadherin [13] and desmogleins (this report) independently. In cell-based assays where both E-cadherin and desmogleins (Dsg2) are present, we can not dissect whether the action of hK7 upon a particular substrate is a primary or secondary effect, or whether both substrates are acted upon concurrently. Based upon our *in vitro *results, we favor the notion that hK7 cleaves each substrate from the cell surface independently, with their combined loss contributing to the morphological changes observed. Other hK7 substrates that have yet to be elucidated, however, may also participate in this process.

## Conclusion

In this report we have shown that the levels of cell-surface resident Dsg1 and Dsg2 are reduced in pancreatic adenocarcinomas compared with normal and chronic pancreatitis tissues. We have also demonstrated that these desmosomal proteins are substrates of the chymotryptic-like protease hK7, which is overexpressed in pancreatic cancer. Using a cell-based system, we have found that expression of hK7 results in a marked increase in the shedding of an 80-kDa soluble fragment of Dsg2 into the conditioned medium of BxPC-3 cells. These results, therefore, provide evidence that hK7 may play a critical role in the invasion of cancer cells in which it is aberrantly expressed by degrading important cell adhesive molecules like desmogleins and E-cadherin [13].

## Competing interests

The authors declare that they have no competing interests.

## Authors' contributions

VCR participated in the design of the study, carried out all of the technical procedures, and performed the statistical analyses. LH performed the immunohistochemical analyses. RH supervised the design and implementation of the study. All authors participated in the preparation of the manuscript and have read and approved the final manuscript.

## Pre-publication history

The pre-publication history for this paper can be accessed here:



## Supplementary Material

Additional file 1 **Desmoglein 1 staining in normal human skin**. As a positive control, immunohistochemistry performed on sections of normal human skin with the Dsg1 antibody used for staining pancreatic tissues revealed basilar and suprabasilar epidermal staining. Click here for file

Additional file 2 **Laminin is not a substrate of hK7 *in vitro***. Thermolysin-activated hK7 (200 ng) was incubated with 1 μg of laminin for the indicated times and the reaction products were separated by SDS-PAGE and visualized by Coomassie staining. Sizes of protein markers are indicated on the left. Click here for file

Additional file 3 **Expression of hK7 in BxPC-3 cells results in an altered cell morphology**. Phase-contrast (*upper*) or rhodamine phalloidin-stained (*lower*) images of hK7-expressing clones BxPC-3/hK7 clone#3 and clone#7 reveal altered cell morphology compared with vector-transfected cells (BxPC-3/Vec). Cells expressing hK7 display reduced cell-cell contacts and a more flattened, less refractive appearance. Click here for file
