# Supplementary figures and images for: Desmoglein 2 is a substrate of kallikrein 7 in pancreatic cancer
Source: BMC Cancer. 2008 Dec 17;8:373. doi: 10.1186/1471-2407-8-373 (PMC2628383; doi:10.1186/1471-2407-8-373)

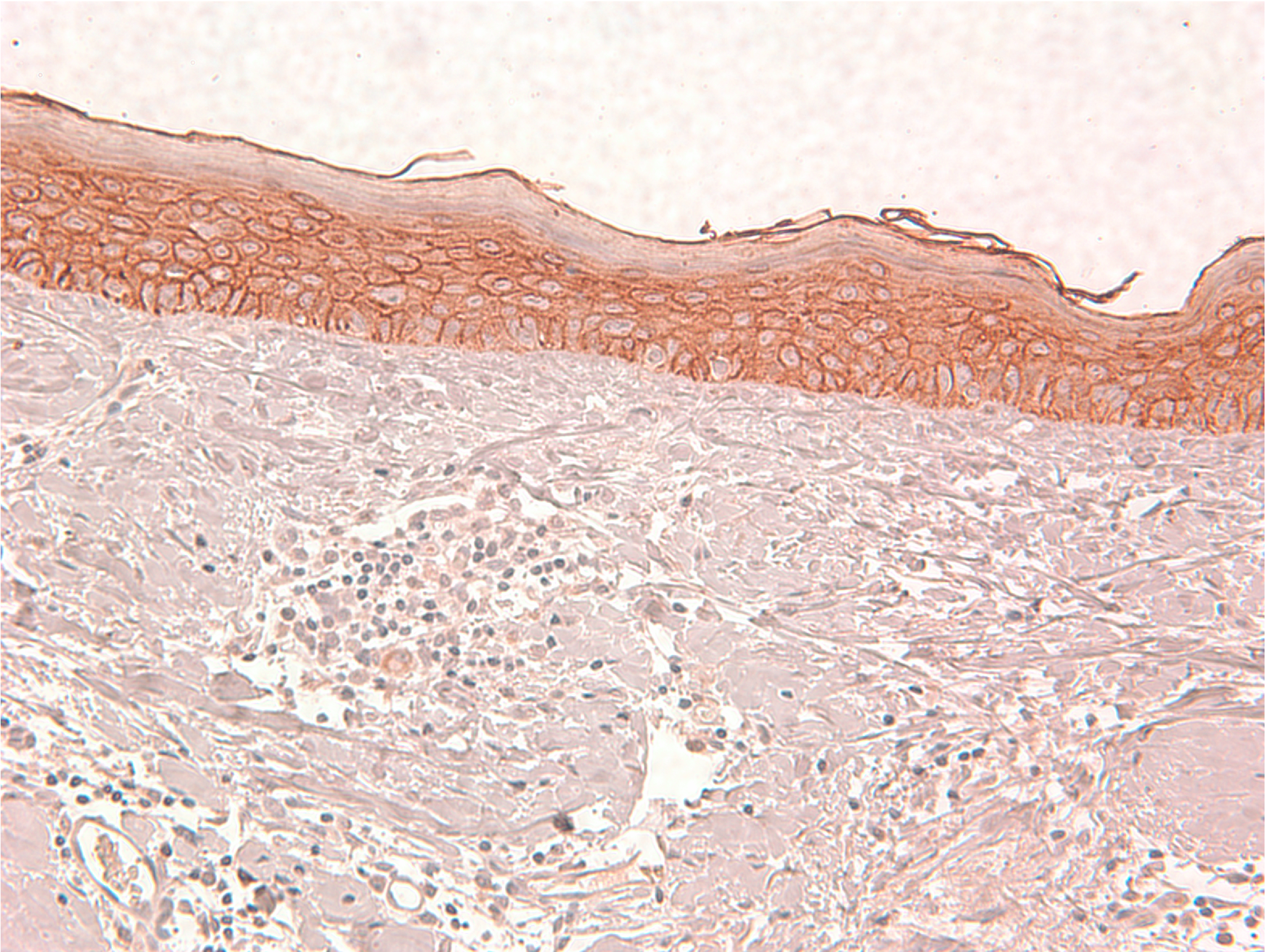

Supplement: Additional file 1 — Desmoglein 1 staining in normal human skin. As a positive control, immunohistochemistry performed on sections of normal human skin with the Dsg1 antibody used for staining pancreatic tissues revealed basilar and suprabasilar epidermal staining. [file 1471-2407-8-373-S1.png]

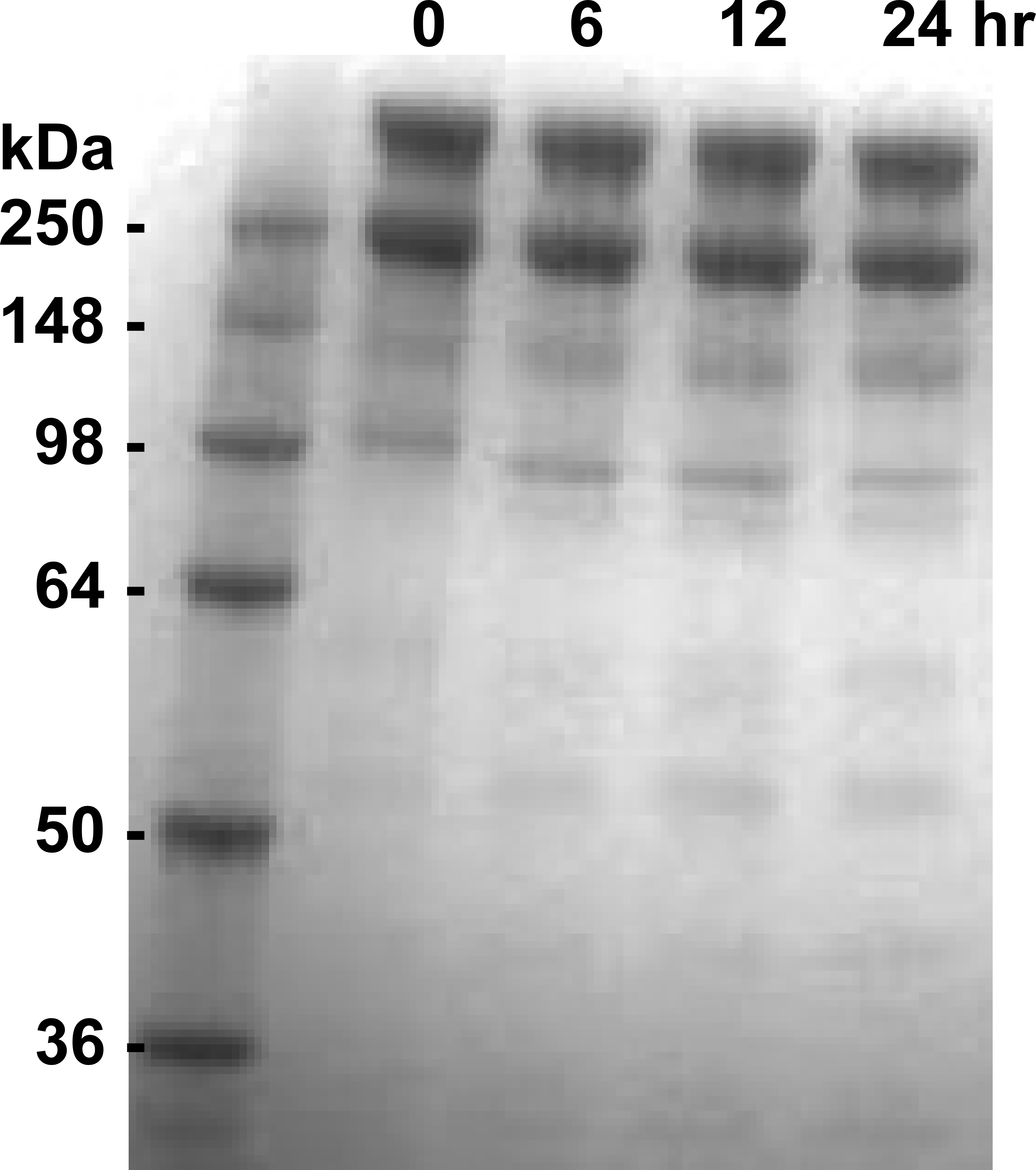

Supplement: Additional file 2 — Laminin is not a substrate of hK7 in vitro. Thermolysin-activated hK7 (200 ng) was incubated with 1 μg of laminin for the indicated times and the reaction products were separated by SDS-PAGE and visualized by Coomassie staining. Sizes of protein markers are indicated on the left. [file 1471-2407-8-373-S2.png]

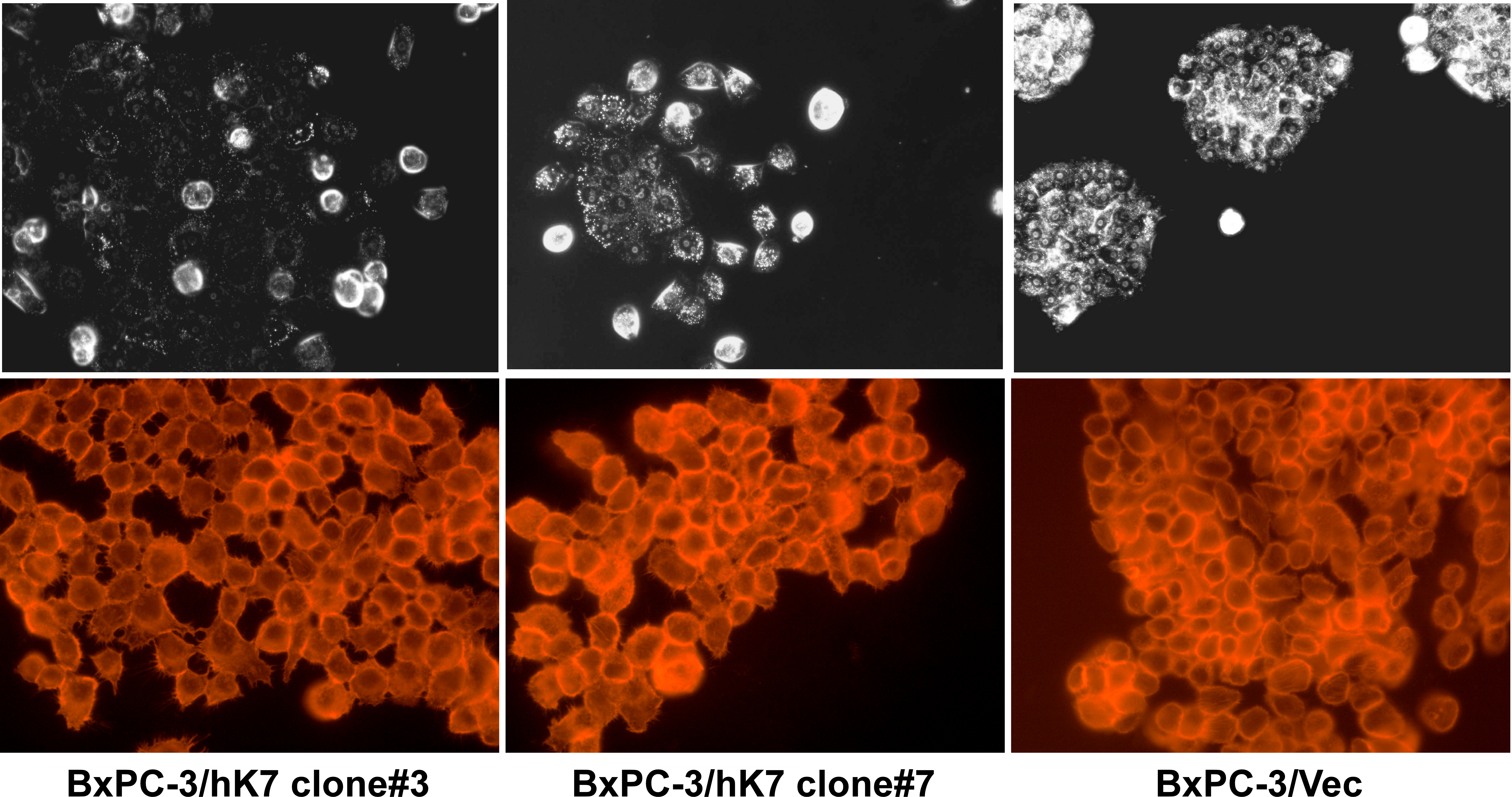

Supplement: Additional file 3 — Expression of hK7 in BxPC-3 cells results in an altered cell morphology. Phase-contrast (upper) or rhodamine phalloidin-stained (lower) images of hK7-expressing clones BxPC-3/hK7 clone#3 and clone#7 reveal altered cell morphology compared with vector-transfected cells (BxPC-3/Vec). Cells expressing hK7 display reduced cell-cell contacts and a more flattened, less refractive appearance. [file 1471-2407-8-373-S3.png]
